# *Xylocopa caerulea* and *Xylocopa auripennis* harbor a homologous gut microbiome related to that of eusocial bees

**DOI:** 10.3389/fmicb.2023.1124964

**Published:** 2023-05-17

**Authors:** Yifan Gu, Wensu Han, Yuquan Wang, Danlei Liang, Jinglin Gao, Yihai Zhong, Shan Zhao, Shijie Wang

**Affiliations:** ^1^Environment and Plant Protection Institute, Chinese Academy of Tropical Agricultural Sciences, Haikou, China; ^2^Sanya Institute of China Agricultural University, Sanya, China; ^3^Department of Entomology, College of Plant Protection, China Agricultural University, Beijing, China; ^4^Bee Industry Technology Research Center, Chinese Academy of Tropical Agricultural Sciences, Haikou, China; ^5^College of Plant Protection, Hainan University, Haikou, China

**Keywords:** carpenter bees, gut microbiota, bacteria symbiosis, *Candidatus Schmidhempelia*, *Bombiscardovia*, eusocial bees, *Bombilactobacillus*

## Abstract

**Background:**

Eusocial bees, such as bumblebees and honey bees, harbor host-specific gut microbiota through their social behaviors. Conversely, the gut microbiota of solitary bees is erratic owing to their lack of eusocial activities. Carpenter bees (genus *Xylocopa*) are long-lived bees that do not exhibit advanced eusociality like honey bees. However, they often compete for nests to reproduce. *Xylocopa caerulea* and *Xylocopa* auripennis are important pollinators of wild plants on Hainan Island. Whether they have host-specific bacteria in their guts similar to eusocial bees remains unknown.

**Methods:**

We targeted the bacterial 16S rRNA V3-V4 region to investigate the diversity of bacterial symbionts in the fore-midgut and hindgut of two carpenter bees, *X. caerulea* and *X. auripennis*.

**Results:**

A maximum of 4,429 unique amplicon sequence variants (ASVs) were detected from all samples, belonging to 10 different phyla. *X. caerulea* and *X. auripennis* shared similar bacterial community profiles, with Lactobacillaceae, Bifidobacteriaceae, and Orbaceae being dominant in their entire guts. *X. caerulea* and *X. auripennis* harbor a highly conserved core set of bacteria, including the genera *Candidatus Schmidhempelia* and *Bombiscardovia*. These two bacterial taxa from carpenter bees are closely related to those isolated from bumblebees. The LEfSe analysis showed that Lactobacillaceae, Bifidobacteriaceae, and the genus *Bombilactobacillus* were significantly enriched in the hindguts of both carpenter bees. Functional prediction suggested that the most enriched pathways were involved in carbohydrate and lipid metabolism.

**Conclusions:**

Our results revealed the structure of the gut microbiota in two carpenter bees and confirmed the presence of some core bacterial taxa that were previously only found in the guts of social bees.

## Introduction

Bees are well-known for their abundant biodiversity, with more than 17,500 identified species (Danforth et al., 2013). Among them, honey bees, bumblebees, and stingless bees are domesticated by humans to provide essential pollination services for crops and fruit trees (Brittain et al., 2013). These bees exhibit social behaviors and have a complete social structure, whereas most other bee species are solitary. Solitary bees have broad geographic distribution, varying body sizes, and diverse foraging preferences, making them irreplaceable pollinators in the ecological environment with flowering plants, even in places where domestic honey bee populations exist (Hedtke et al., 2013). However, the ecosystem roles of solitary bees have often been overlooked by researchers. Most studies have focused on eusocial bees, such as the honey bee *Apis mellifera*, while the study of solitary bees has been scarcely investigated.

The microbiota represents a vital indicator of the fitness and health of numerous insect species. Moreover, microorganisms play a critical role in many interactions between insect hosts and their habitats. For example, in the camellia weevil *Curculio chinensis*, the microbiota was responsible for tea saponin degradation in the insect's feeding (Zhang et al., 2020). Even though symbionts can be beneficial for their host, they can also bring negative effects to some insects. Among arthropods, *Wolbachia* spp. has been identified as a bacteria symbiont that distorts the reproductive cells, thereby enhancing its maternal transmission into subsequent progenies. Consequently, it has been deemed a novel pest biocontrol bacterium (Ali et al., 2018). The hispid leaf beetle *Octodonta nipae* is naturally infected with *Wolbachia*, which has been identified as an obligate endosymbiont present in all life stages, body parts, and tissues that were tested (Ali et al., 2019). Similar to other insects, the bacteria symbiont of honey bees plays a critical role in their survival. Massive losses of honey bee colonies and the decline of many bumblebee species have elicited global concern in recent decades (Lee et al., 2015; Hammer et al., 2021). A large body of evidence suggests that gut microbiota is critical in maintaining bee health (Engel et al., 2016; Jones et al., 2017; Zheng et al., 2018). Previous studies have shown that honey bees and bumblebees harbor distinctive core gut bacterial communities that are transmitted through social behaviors such as oral trophallaxis and fecal–oral pathway (Khan et al., 2020; Hammer et al., 2021). The abundance and species of core bacteria have been found to be remarkably stable, showing little effect across various habitats (Anderson et al., 2015; Kwong and Moran, 2016; Hammer et al., 2021). Host-specific bacteria have diverse functions in the digestion and absorption of nutrients, as well as in defending against pathogen colonization and reinforcing the host's immune system (Zheng et al., 2017; Kešnerová et al., 2019; Ribière et al., 2019). However, most studies have focused on interactions between gut bacteria and social bees, such as *A. mellifera* and *Bombus terrestris*, and the study of solitary bees has been scarcely investigated.

Carpenter bees belong to one taxon of wild bees (genus: *Xylocopa*) and are known for their large body size. They play a crucial role in crop pollination due to their greater pollination efficiency compared to honey bees in some cultivated large-flower plants such as passion fruit *Passiflora edulis* (Junqueira and Augusto, 2016; Alberoni et al., 2019; de Farias-Silva and Freitas, 2021). The *Xylocopa* genus comprises about 470 species worldwide (Michener, 2000) and 40 species mainly described in tropical and subtropical China (Wu, 2000). The social behavior of the genus *Xylocopa* is not well-understood. Although incipient social behaviors have been observed in some species in the wild, knowledge is lacking for most species (Handy et al., 2023). Female *Xylocopa* excavates their nests in dry plant tissues, such as trees, dead trunks, and bamboo canes (Junqueira et al., 2012), and lay eggs in cells with pollen and nectar (Keasar et al., 2007). Unlike social bees, nests of *Xylocopa* are often reused for several years (Yamamoto et al., 2014). Newly emerged female *Xylocopa* leave their old nest and find other abandoned nests to reproduce during the nesting seasons. Nest reusing is more common in an environment of limited nesting materials. In addition to the aforementioned characteristics, fighting between female *Xylocopa* for nests can also result in the reuse of old nests (He et al., 2013). Nest reusing is generally a common behavior in the genus *Xylocopa*.

At present, the composition of microbiota in *Xylocopa* species is rarely studied, with most of the microbiota species of these bees remaining unclear. However, a few available studies suggest that carpenter bees have consistent relationships with specific bacterial taxa. *Xylocopa micans, Xylocopa mexicanorum*, and *Xylocopa tabaniformis parkinsoniae*, which are carpenter bees from central Texas, were found to share similar gut bacterial communities, including Bifidobacteriaceae, Orbaceae, Lactobacillaceae, Pseudomonadaceae, and Enterobacteriaceae. Meanwhile, *Xylocopa virginica* had a distinct microbiota dominated by the genus *Bombilactobacillus*, a group of bacteria abundant in the guts of eusocial bees (Holley et al., 2022). In *Xylocopa tenuiscapa*, the diversity of bacteria in the foregut and hindgut were found to be different, and certain species, such as *Gilliamella, Lactobacillus*, and *Bifidobacterium*, were found to be related to those found in honey bees (Subta et al., 2020).

In the present study, we investigated the gut bacteria of two *Xylocopa* species (*X*. *caerulea* and *X*. *auripennis*) from different ecological environments. *X. caerulea* is found in Jianfengling National Forest Park of Hainan (~838 m above sea level), which is a tropical forest area rich in natural resources and one of the best protected in the region. On the other hand, *X. auripennis* inhabits mountainous villages with ample farmland (~650 m above see level). Symbionts are spatially organized within specific gut regions (Zheng et al., 2017, 2018). Here, we used 16S rDNA sequencing to investigate the gut bacterial communities in different parts of the intestinal tract of two *Xylocopa* species. Our results showed that these two carpenter bee species have consistent gut communities and specific gut symbionts that are commonly found in bumblebees. This finding provides novel insights into the symbiotic gut communities of solitary bees.

## Materials and methods

### Sample collection

**Figure 1:**
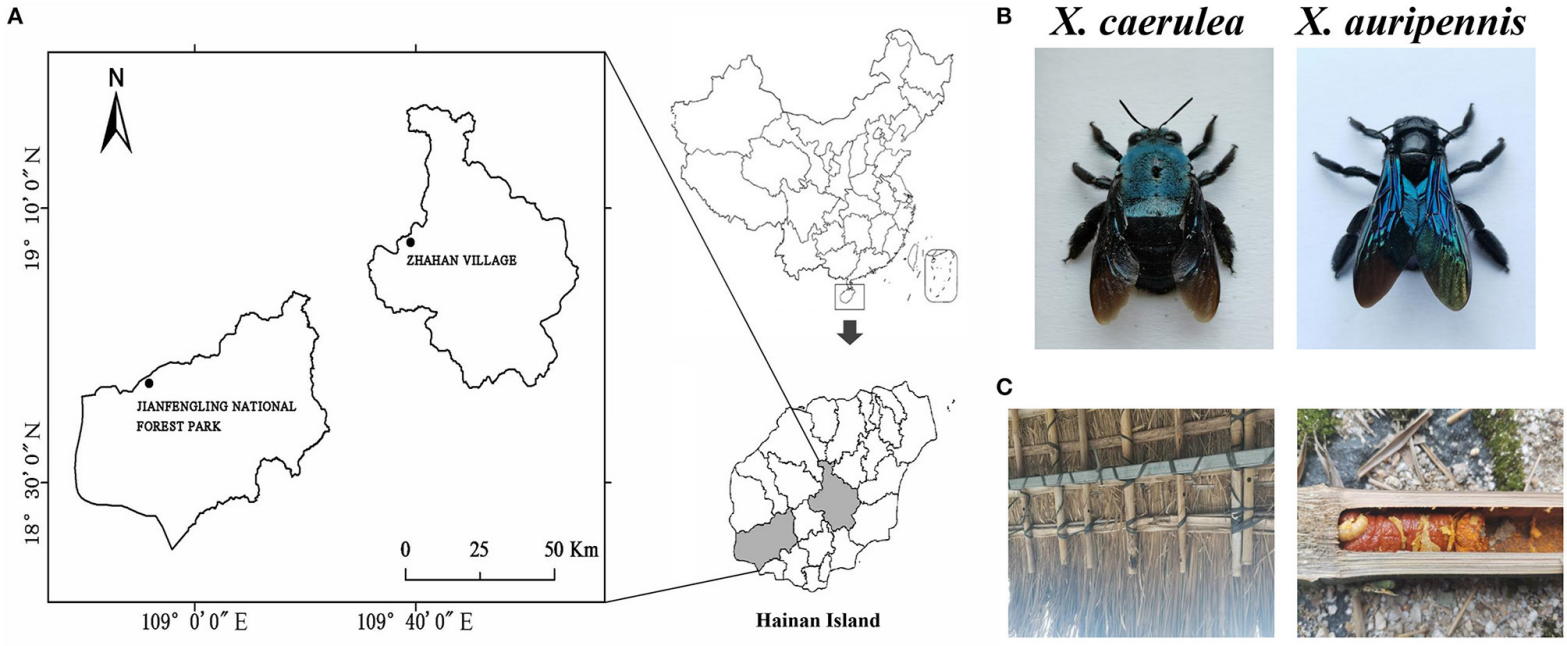
Collecting locations of carpenter bees. **(A)** Jianfengling National Forest Park in Hainan (18°44′28^′′^N, 108°51′39^′′^E) and Zhahan village of Hongmao town in Qiongzhong Li and Miao Autonomous County (19°4′58^′′^N, 109°38′59^′′^E), **(B)** *Xylocopa caerulea* and *Xylocopa auripennis*, and **(C)** nest and larvae of *X. auripennis*.

Pollinating carpenter bee samples (*X. caerulea* and *X. auripennis*) were used for gut microbial composition analysis. Six *X. caerulea* were collected from the Jianfengling National Forest Park in Hainan (18°44′28^′′^N, 108°51′39^′′^E) and six *X. auripennis* were collected by sweep nets in April 2022 in Hainan Province from Zhahan village of Hongmao town, Qiongzhong Li, and Miao Autonomous County (19°4′58^′′^N, 109°38′59^′′^E; Figure 1). The collected carpenter bees were transported to the laboratory in a bubble chamber with ice packs.

### Insect dissection and DNA extraction

To extract bacterial DNA, *X. caerulea* and *X. auripennis* were dissected immediately upon arrival at the laboratory. After freezing the live carpenter bees in a −20°C refrigerator, each bee was washed three times with 75% alcohol and then several times with sterile water. The whole gut of each carpenter bee was carefully removed using sterile tweezers and scissors. Then the gut was divided into two parts: hindgut and fore-midgut (including crop), which were immediately placed in a 1.5 ml centrifuge tube, respectively, frozen in liquid nitrogen, and stored at −80°C refrigerator until further analysis. Six replicates of the dissected intestinal tract of the carpenter bee (one individual/sample) were processed for DNA extraction.

DNA extraction was carried out using the E.Z.N.A.^®^Stool DNA Kit (D4015, Omega, Inc., USA) according to the manufacturer's instructions. Nuclear-free water was used as a blank. The total DNA was eluted in 50 μl of elution buffer (Tris–hydrochloride buffer, pH 8.0, containing 1.0 mM EDTA) and stored at −80°C until usage for the PCR.

### 16S rRNA amplification

For each gut DNA sample, PCR was conducted for the V3–V4 region of bacterial 16S rRNA gene using the primer set 341F (5′-CCTACGGGNGGCWGCAG-3′) and 805R (5′-GACTACHVGGGTATCTAATCC-3′). The 5′ ends of the primers were tagged with specific barcodes per sample and sequencing universal primers. PCR amplification was performed in a total volume of 25 μl of reaction mixture containing 25 ng of template DNA, 12.5 μl of PCR Premix, 2.5 μl of each primer, and PCR-grade water to adjust the volume. The PCR conditions are divided into two steps: initial denaturation at 95°C for 5 min, followed by 25 cycles of denaturation at 95°C for 30 s, annealing at 50°C for 30 s, and extension at 72°C for 40 s, and then the final extension at 72°C for 7 min. In the second step, there was an initial denaturation at 98°C for 30 s, followed by seven cycles of denaturation at 98°C for 10 s, annealing at 65°C for 30 s, and extension at 72°C for 30 s, then the final extension at 72°C for 5 min.

After amplification, the PCR products were confirmed with imaging of 2% agarose gel electrophoresis. Throughout the DNA extraction process, ultrapure water was used instead of a sample DNA to exclude false-positive PCR results as a negative control.

### Library preparation and sequencing

The PCR products underwent purification using AMPure XT beads (Beckman Coulter Genomics, Danvers, MA, USA) and quantification using Qubit (Invitrogen, USA). The amplicon pools were prepared for sequencing, and the size and quantity of the amplicon library were assessed using an Agilent 2100 Bioanalyzer (Agilent, USA) and with the library Quantification Kit for Illumina (Kapa Biosciences, Woburn, MA, USA), respectively. The libraries were sequenced on the NovaSeq PE250 platform. Sequencing and bioinformatics analyses were performed by a commercial company (Biotree, Shanghai, China).

### Sequence analysis

Paired-end reads were assigned to samples based on their unique barcode and primer sequence. The paired-end reads were merged using FLASH software. Quality filtering was performed on the raw reads under specific filtering conditions to obtain high-quality clean tags according to the fqtrim (v0.94). Chimeric sequences were filtered using V search software (v2.3.4). After dereplication using DADA2, we obtained a feature table and associated sequences. After that, amplicon sequence variants (ASVs) were clustered and annotated at a 97% similarity threshold. Sequences with ambiguous, chloroplast, or mitochondrion assignments were removed. Alpha diversity and beta diversity were calculated by normalizing them to the same sequences randomly. According to the SILVA (release 132) classifier, feature abundance was normalized using the relative abundance of each sample. Alpha diversity was applied in analyzing the complexity of species diversity for a sample through five indices, including Chao1, Observed species, Goods coverage, Shannon, and Simpson.

### Bacterial phylogenetic reconstruction

Amplicon sequence variants were subjected to the BLAST approach against the NCBI nucleotide collection database for phylogenetic construction. The phylogenetic tree was built based on the sequence alignment using the neighbor-joining (NJ) algorithm in the software of Mega X program (Kumar et al., 2018). The reliability of the branching was tested by bootstrap resampling (1,000 pseudo-replicates).

### Putative functional profiling

Phylogenetic Investigation of Communities by Reconstruction of Unobserved States (PICRUSt2; https://github.com/picrust/picrust2) was adopted for the functional prediction of gut microbiota (Douglas et al., 2020). Functional community profiling was predicted based on the bacterial 16S rDNA gene ASVs associated with different parts of the intestinal tract. Sequenced prokaryotic genomes of 16S rDNA gene sequences were linked to the Kyoto Encyclopedia of Genes and Genomes (KEGG) ortholog for functional annotation.

### Statistical analysis

In this study, QIIME2 was used to compare sample complexity and diversity. The basic analysis of ASVs was performed, including the generation of a Venn diagram of ASVs distribution and ASV cluster analysis. Principal coordinate analysis (PCoA) was performed on unweighted UniFrac distance matrices to study the similarities of differences in sample community composition. The differences in the community structure of the gut microbiota at five levels (ranging from phylum to genus) between two species and hindgut and fore-midgut were analyzed using non-parametric factorial Mann–Whitney *U*-test (*P* < 0.05) and estimated LDA score using linear discriminant analysis effect size (LEfSe), with an LDA threshold of ≥3. PCoA analysis was calculated and visualized using R statistical software (Lockstone, 2011). The STAMP software (version 2.1.3) was employed to identify the significant differences in the relative abundance of predicted gene proportion between the fore-midguts and hindguts of two carpenter bee species (Welch's *t*-test, *P* < 0.05).

## Results

### Bacterial diversity estimation

**Figure 2:**
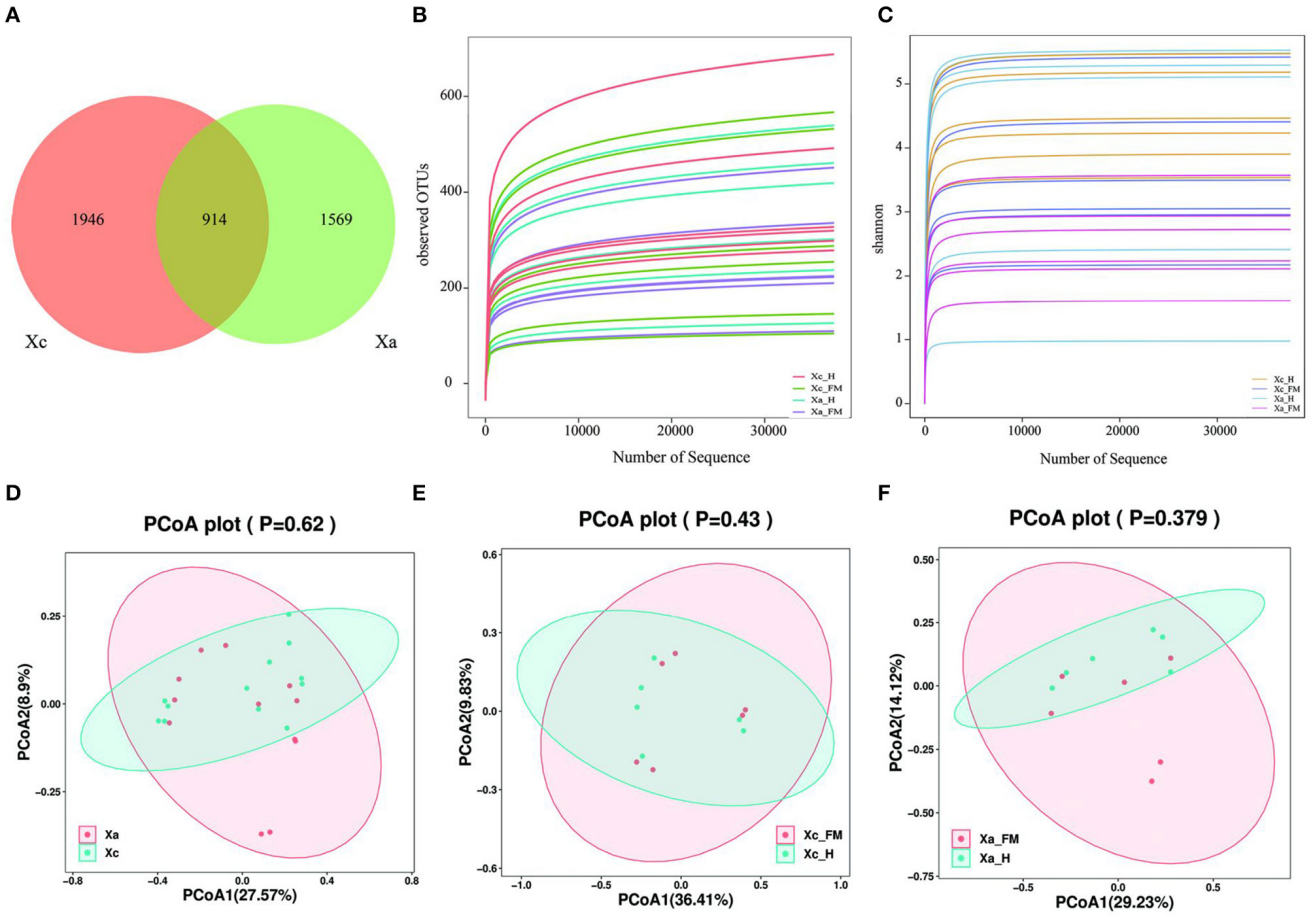
Gut bacterial diversity of carpenter bees from different locations in Hainan Island of China. **(A)** Venn diagram of the distribution of ASVs in the two carpenter bees. **(B)** Rank-abundance curves. **(C)** Shannon index rarefaction curves. **(D–F)** Unweighted UniFrac principal component analysis (PCoA) estimates for the gut bacteria of **(D)** *X. caerulea* and *X. auripennis* **(E)** fore-midgut and hindgut of *X. caerulea* **(F)** fore-midgut and hindgut of *X. auripennis*. Xa, *X. auripennis*; Xc, *X. caerulra*. Xc_FMG, the fore-midgut of *X. caerulra*; Xc_HG, the hindgut of *X. caerulra*; Xa_FMG, the fore-midgut of *X. auripennis*; Xa_HG, the hindgut of *X. auripennis*. The same is as follows.

A total of 1,695,348 valid sequences of the16S rDNA gene were acquired from six *X. caerulea* and six *X. auripennis* samples after stringent quality checking (Supplementary Table S1). A maximum of 4,429 unique amplicon sequence variants (ASVs) were clustered based on a 97% similarity cutoff. Among these ASVs, there were 1,946 ASVs unique to *X. caerulea* and 1,569 ASVs unique to *X. auripennis*. The shared ASVs (914) accounted for 20.64% of total ASVs. These ASVs were presented in a Venn diagram (Figure 2A) and classified into 42 phyla, 126 classes, 258 orders, 411 families, 821 genera, and ~1,088 species. The rarefaction curves of bacterial diversity estimators (observed OTUs and Shannon) for all samples reached a plateau phase, indicating that most microbial species had been captured in all samples (Figures 2B, C). Meanwhile, Good's coverage was used to check the completeness of sequencing. The results showed that the coverage of each sample was above 99.99%, indicating that most species in the sample were identified. The alpha diversity indices were estimated to uncover the bacterial diversity (Simpson and Shannon), species richness (Chao1 and observed OTUs), and bacterial coverage (Good′s coverage) (Table 1). Based on these indices, no significant differences were detected between *X. caerulea* and *X. auripennis* comparisons. Similarly, there was no significant difference between the hindgut and fore-midgut of *X. caerulea* and *X. auripennis*. The beta diversity estimates were calculated by computing unweighted UniFrac and visualized by principal coordinates analysis (PCoA). The results indicated that the gut bacterial communities of *X. caerulea* were not significantly different from those of *X. auripennis* (Figure 2D). Moreover, the bacterial communities in the hindgut were not significantly different from the fore-midgut in *X. caerulea* or *X. auripennis* (Figures 2E, F).

**Table 1:** Alpha diversity indices of the gut microbiota of carpenter bees *X. caerulea* and *X. auripennis*.

**Group**	**Diversity indices (mean** ±**standard deviation)**				
	**Observed OTUs**	**Shannon**	**Simpson**	**Chao1**	**Goods coverage (%)**
Xc	370.50 ± 190.51	4.02 ± 1.04	0.80 ± 0.11	381.70 ± 195.73	>99.99
Xa	317.00 ± 146.88	3.33 ± 1.62	0.64 ± 0.27	327.00 ± 153.74	>99.99
Xc_FMG	324.50 ± 184.86	3.57 ± 1.05	0.76 ± 0.12	331.87 ± 189.65	>99.99
Xc_HG	413.67 ± 164.78	4.46 ± 0.68	0.84 ± 0.08	426.38 ± 176.69	>99.99
Xa_FMG	285.17 ± 124.34	2.53 ± 0.63	0.56 ± 0.17	286.35 ± 124.68	>99.99
Xa_HG	364.00 ± 152.14	4.13 ± 1.78	0.73 ± 0.30	364.25 ± 152.18	>99.99

Xa, *X. auripennis*; Xc, *X. caerulea*. Xa and Xc stand for the sequence data of whole guts in *X. caerulea* and *X. auripennis*, respectively. Xc_FMG, the fore-midgut of *X. caerulea*; Xc_HG, the hindgut of *X. caerulea*; Xa_FMG, the fore-midgut of *X. auripennis*; Xa_HG, the hindgut of *X. auripennis*. The same is as follows. There were no significant differences detected between Xa and Xc, Xc_FMG and Xc_HG, Xa_FMG, and Xa_HG. Data (mean ±SD) were analyzed by Mann–Whitney U-test.

### Relative abundance of gut bacterial communities

**Figure 3:**
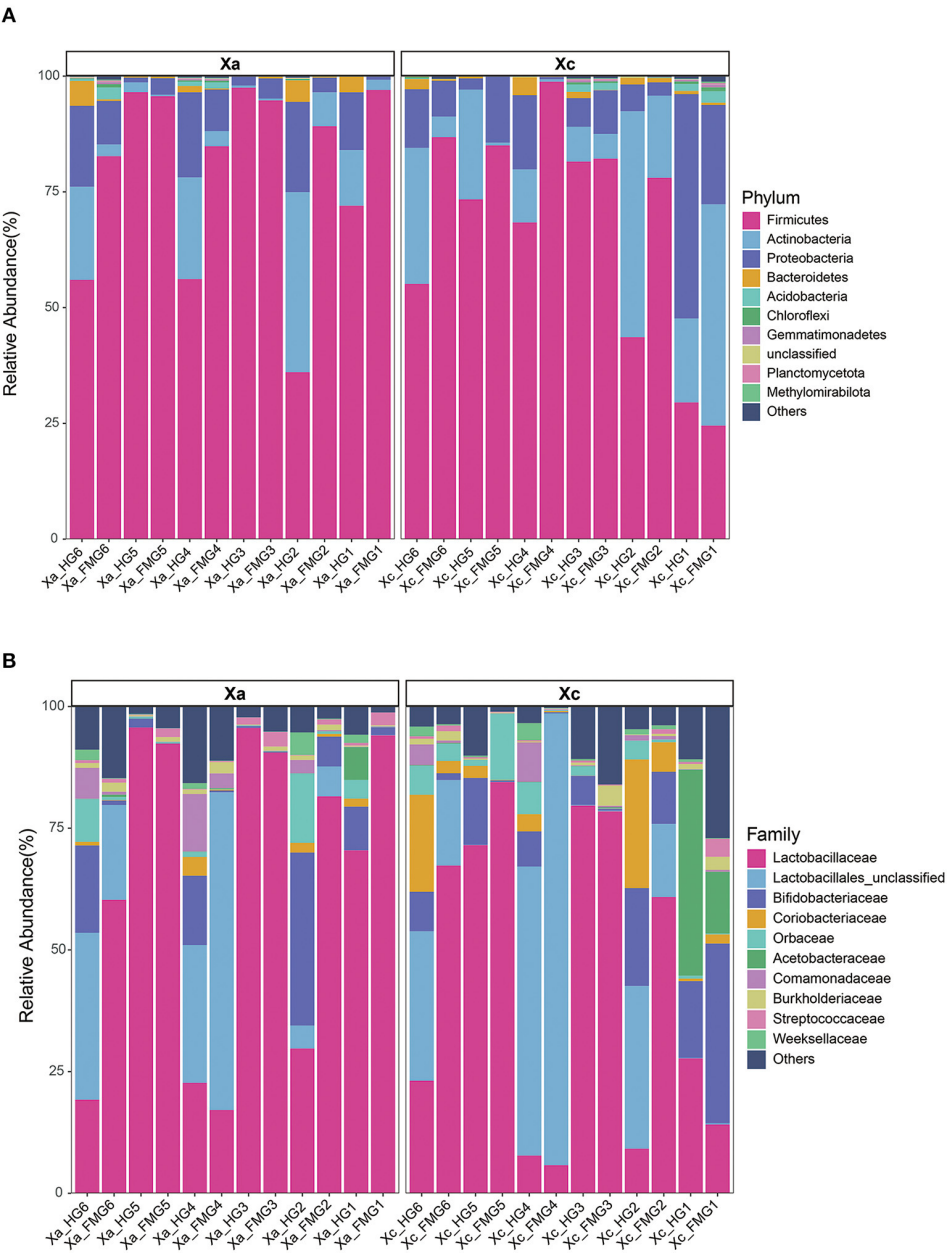
The gut bacterial composition of the carpenter bees on **(A)** phylum and **(B)** family. The top 10 taxa in abundance were shown in the bar charts. Each color represents a species, and the height of the color block indicates the proportion of the species in relative abundance. Other species are incorporated as “Others” shown in the diagram.

The two carpenter bee species *X. caerulea* and *X. auripennis* shared similar bacterial community profiles. At the phylum level (Figure 3A), Firmicutes was the dominant phylum, with a relative abundance of 67.26% in *X. caerulea* and 79.88% in *X. auripennis*. Actinobacteria, Proteobacteria, and Bacteroidota were also dominant in both species, with relative abundances of 17.94 and 9.29%, 12.32 and 8.41%, 0.99 and 1.37%, respectively.

At the family level (Figure 3B), *X. caerulea* and *X. auripennis* also shared similar bacterial community profiles. Lactobacillaceae was the most abundant family (44.17 and 64.12%), followed by Lactobacillales unclassified (20.80 and 13.18%), Bifidobacteriaceae (10.08 and 7.35%), Coriobacteriaceae (5.30 and 0.76%), Orbaceae (3.19 and 2.50%), and Acetobacteraceae (0.65 and 4.64%) in *X. caerulea* and *X. auripennis*, respectively.

**Figure 4:**
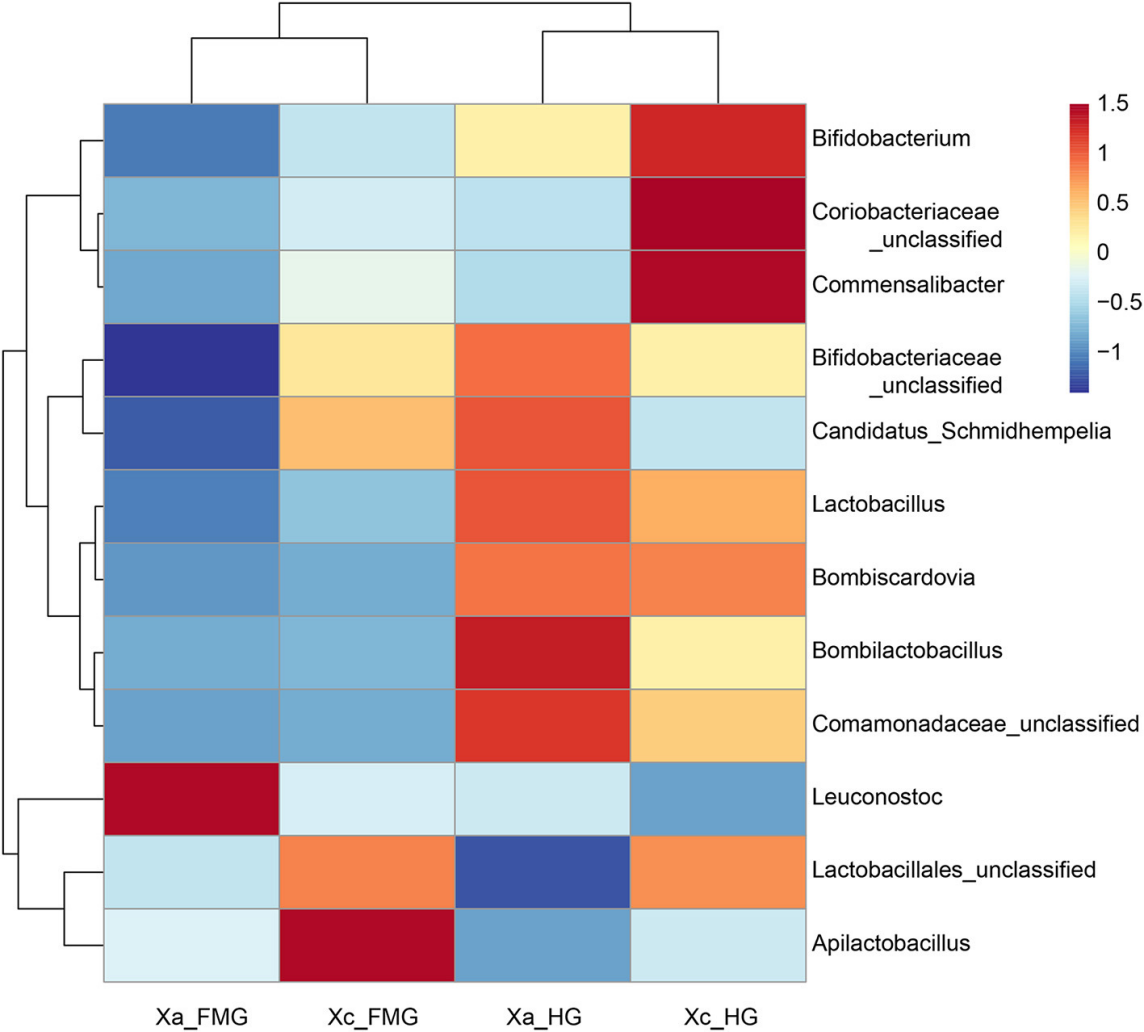
Heatmap of the 12 most abundant genera in the bacterial community at the genus level. Dendrograms of hierarchical cluster analysis grouping genera and samples are shown on the left and at the top, respectively.

At the genus level, the top 12 genera in relative abundance (>1%) were *Leuconostoc, Lactobacillales unclassified, Apilactobacillus, Bombilactobacillus, Bifidobacteriaceae unclassified, Lactobacillus, Coriobacteriaceae unclassified, Commensalibacter, Candidatus Schmidhempelia, Comamonadaceae unclassified, Bifidobacterium*, and *Bombiscardovia*. A clustering analysis of species abundance similarity among the top 12 genera was performed and presented in a heat map (Figure 4). In the whole guts, *Leuconostoc* was 2.21-fold more abundant in *X. auripennis* (42.87%) compared to *X. caerulea* (19.40%), and *Apilactobacillus* was 0.35-fold abundant in *X. auripennis* (5.18%) compared to *X. caerulea* (14.92%). *Candidatus Schmidhempelia* and *Bombiscardovia* were first identified in carpenter bees and their relative abundances in *X. caerulea* and *X. auripennis* were similar. The proportion of *Candidatus Schmidhempelia* and *Bombiscardovia* was 1.66% and 1.37% in *X. caerulea* and 1.47% and 1.33% in *X. auripennis*, respectively.

### Comparison of the bacterial community between fore-midgut and hindgut

**Figure 5:**
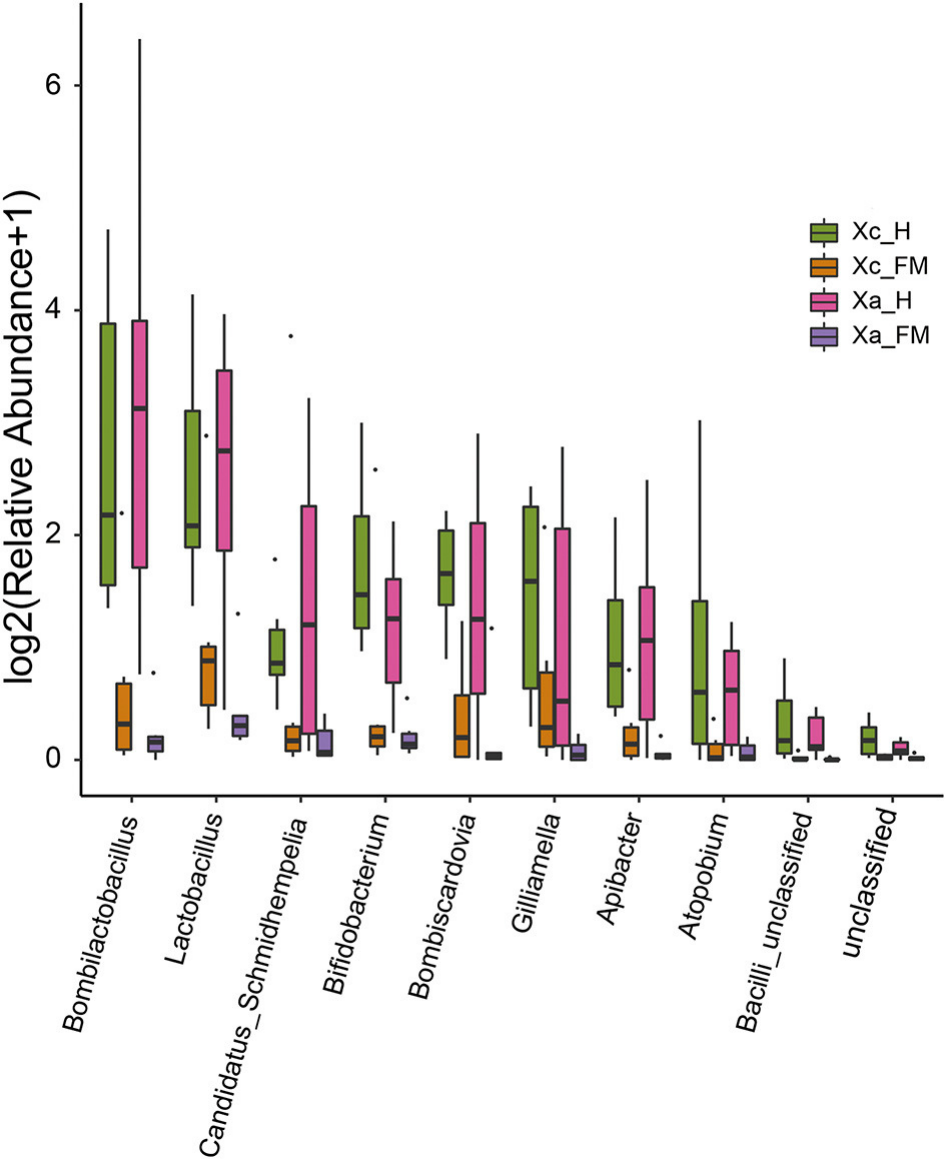
Comparison of the relative abundance of bacterial sequences at the genus level between fore-midguts and hindguts of two carpenter bees. The data were analyzed by the Kruskal–Wallis test (*P* < 0.05), and all the species with *P* < 0.05 are shown in the figure.

The relative abundance difference of bacterial sequences at the genus level between the fore-midgut and hindgut of two carpenter bees was analyzed (Figure 5). The results showed that the abundance of *Bombilactobacillus, Lactobacillus, Candidatus Schmidhempelia, Bifidobacterium, Bombiscardovia, Gilliamella, Apibacter, Atopobium*, and *Bacilli unclassified* in the hindgut was significantly higher than that detected in the fore-midgut.

**Figure 6:**
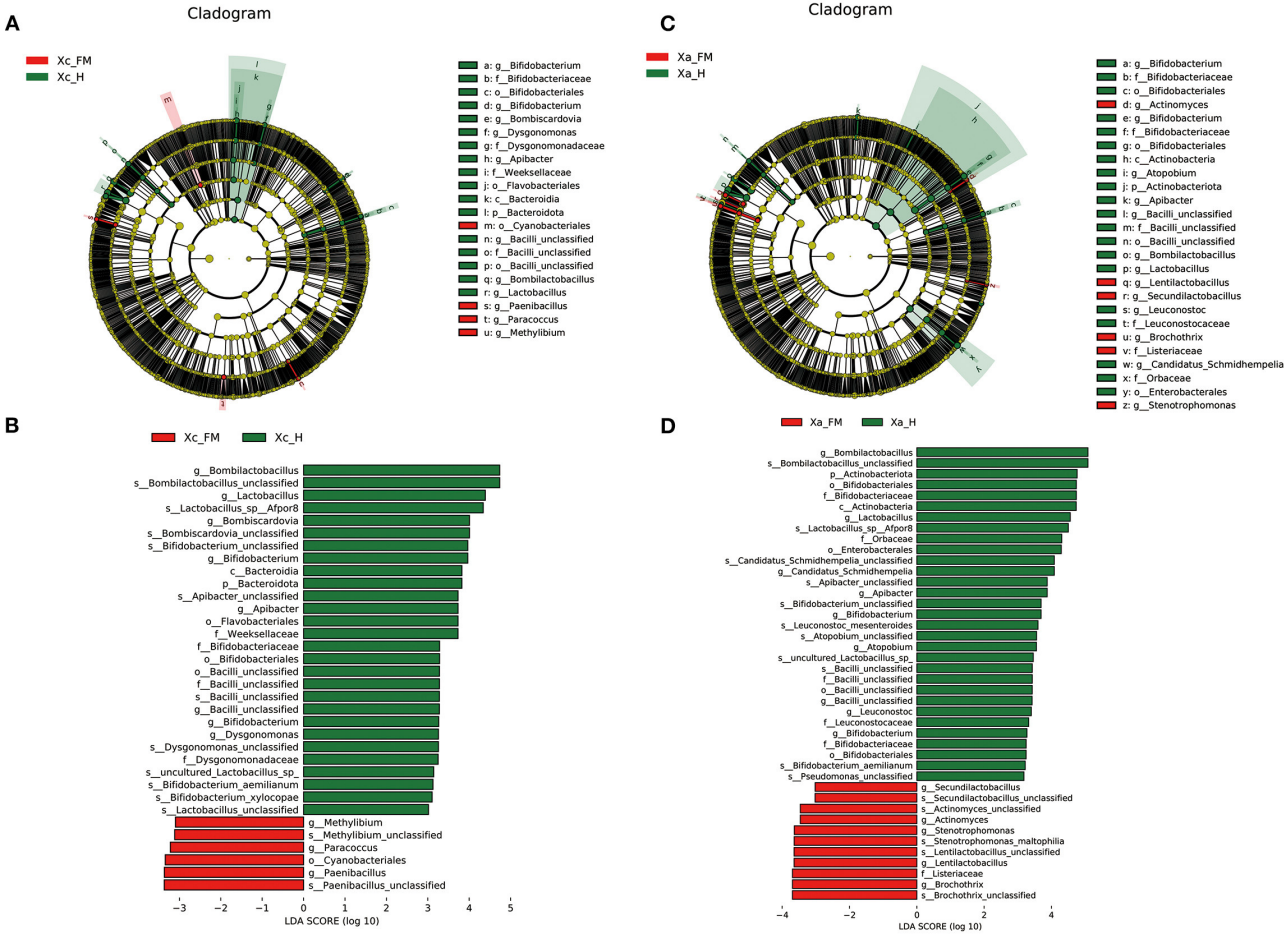
LEfSe analysis showing composition differences in the gut bacteria of fore-midguts and hindguts in two carpenter bees, **(A, B)** *X. caerulea* and **(C, D)** *X. auripennis*. Biomarker taxa are highlighted by colored circles and shaded areas. Each circle's diameter reflects the abundance of taxa in the community. Differential bacterial taxa are marked by lowercase letters. The threshold value of the LDA score was set to 3, and an LDA score of >3 was considered significant.

Linear discriminant analysis effect size analysis confirmed abundance differences of specific taxa between the hindgut and fore-midgut. In the *X. caerulea*, LEfSe analysis revealed that Bacteroidota (phylum), Bacteroidia (class), Flavobacteriales and Bifidobacteriales (order), Weeksellaceae, Dysgonomonadaceae, Bifidobacteriaceae, and Bacilli unclassified (family), *Bombilactobacillus, Lactobacillus, Bombiscardovia, Bifidobacterium, Apibacter, Bacilli unclassified*, and *Dysgonomonas* (genera) were predominant in the hindgut, while *Cyanobacteriales* (order), *Paenibacillus, Paracoccus*, and *Methylibium* (genera) were predominant in the fore-midgut (Figures 6A, B). In the *X. auripennis*, LEfSe analysis identified that Enterobacterales and Bifidobacteriales (order), Orbaceae and Bifidobacteriaceae (family), *Candidatus Schmidhempelia, Bifidobacterium, Bombilactobacillus, Lactobacillus, Apibacter*, and *Atopobium* (genera) were rich in the hindgut, while *Brochothrix, Lentilactobacillus, Stenotrophomonas, Actinomyces*, and *Secundilactobacillus* (genera) were rich in the fore-midgut (Figures 6C, D). Notably, we found that most bacteria enriched in the hindgut of carpenter bees were host-specific bacteria previously known only to bumblebees and honey bees.

### Phylogenetic analyses

**Figure 7:**
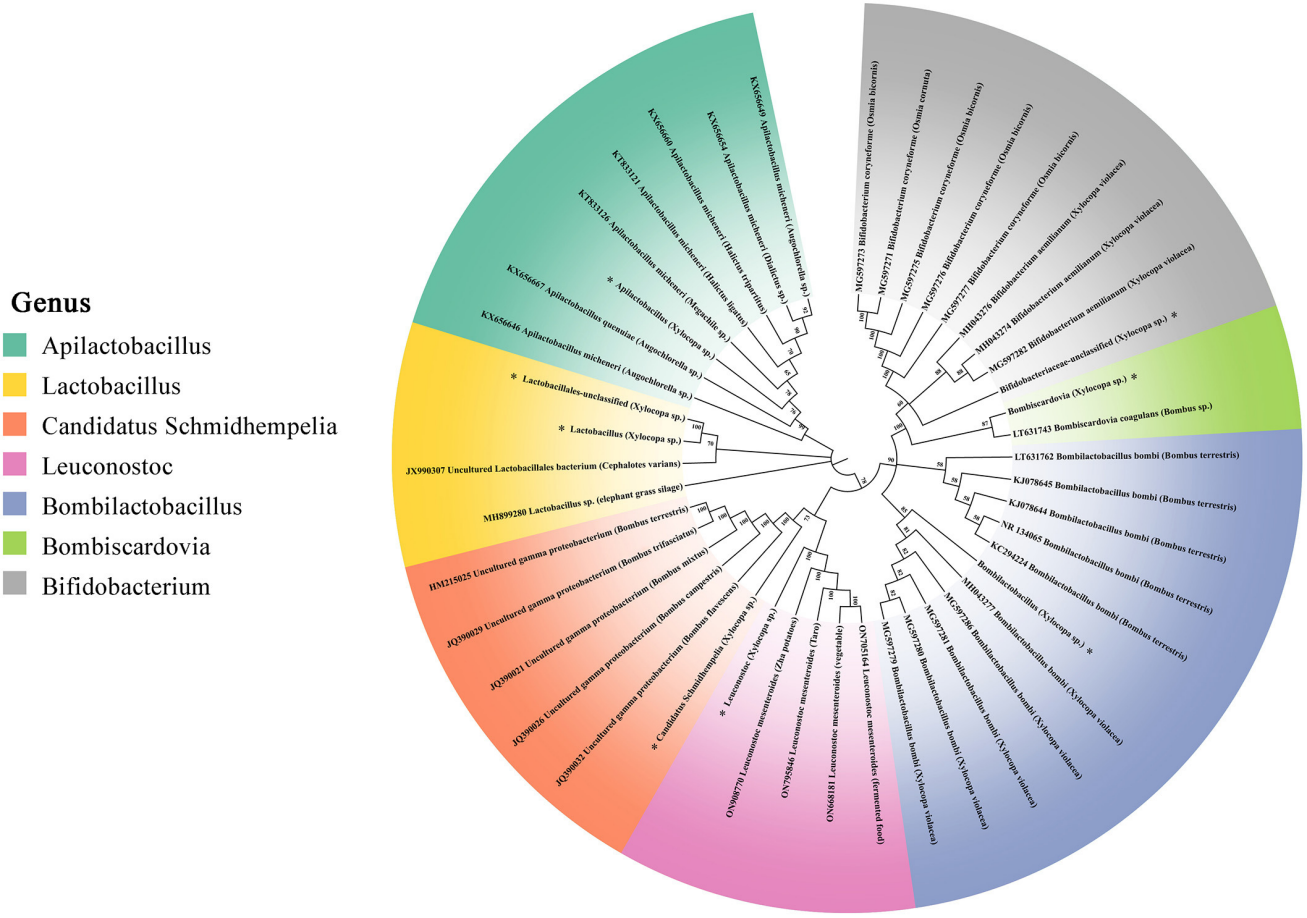
16S rRNA gene phylogenies of bacteria from *Apilactobacillus, Lactobacillus, Candidatus Schmidhempelia, Leuconostoc, Bombilactobacillus, Bombiscardovia*, and *Bifidobacterium*. Phylogenies were inferred by maximum likelihood. ASVs sequenced from *X. caerulea* and *X. auripennis* are represented by asterisks. Host insects are in parentheses.

In Figure 7, the phylogenetic tree of three non-core and four core gut bacteria within carpenter bees-associated ASVs is denoted by an asterisk. *Apilactobacillus* from *Xylocopa* clustered closely with *Apilactobacillus micheneri* and *Apilactobacillus quenuie*, which were isolated from various bees including *Augochlorella* sp, *Dialictus* sp, *Halictus* sp, and *Megachile* sp. Two *Lactobacillus* from *Xylocopa* clustered closely with *Cephalotes*. *Candidatus Schmidhempelia* from *Xylocopa* clustered closely with five uncultured gamma proteobacterium isolated from *Bombus* sp, which were identified in the previous study and renamed *Candidatus Schmidhempelia* (Martinson et al., 2014). *Leuconostoc* from *Xylocopa* clustered with *Leuconostoc mesenteroides*, commonly found in vegetables and fermented food such as potatoes and taros. *Bombilactobacillus* from *Xylocopa* first clustered closely with *Bombilactobacillus bombi* isolated from *Xylocopa violacea*, then with *Bombilactobacillus bombi* isolated from *B. terrestris*. *Bombiscardovia* from *Xylocopa* clustered with *Bombiscardovia coagulans* isolated from *Bombus* sp. *Bifidobacteriaceae unclassified* from *Xylocopa* clustered with *Bifidobacterium aemilianum* and *Bifidobacterium coryneforme* from *X*. *violacea* and *Osmia bicornis*.

### Functional prediction of the gut microbiota

To better understand the important role of the gut microbiota of carpenter bees, we used PICRUSt2 software to predict the compositions of functional genes in samples based on the 16S rDNA sequencing data. The predicted functions were closely related to genetic information processing, cellular processes, organismal systems, environmental information processing, and human diseases and metabolism.

**Figure 8:**
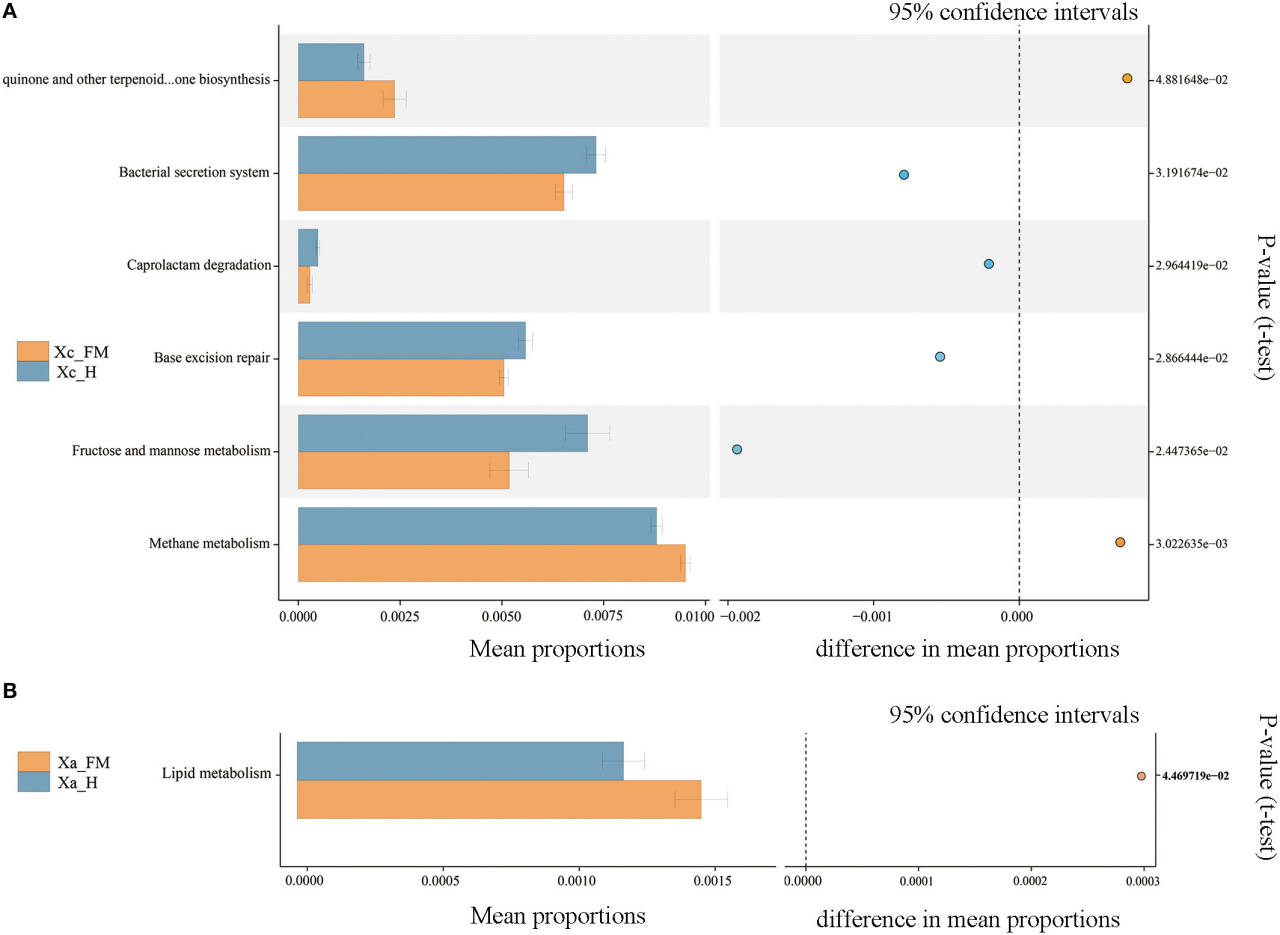
Functions of gut bacterial community predicted by PICRUSt. Significantly different KEGG pathways (level 3) were detected between the fore-midguts and hindguts of *X. caerulea* **(A)** and *X. auripennis* **(B)**.

The functional profile between the fore-midgut and hindgut of *X. caerulea* exhibited significant differences. In the hindgut tract, functions related to the bacterial secretion system, base excision repair, fructose and mannose metabolism, and caprolactam degradation were significantly higher than those in the fore-midgut. In contrast, ubiquinone and other terpenoid–quinone biosynthesis and methane metabolism were significantly lower than those in the fore-midgut (Figure 8A). In the hindgut tract of *X. auripennis*, only lipid metabolism was significantly lower than those in the fore-midgut (Figure 8B).

## Discussion

Carpenter bees are a type of plant pollinator that is covered in thick fur clumps and has a larger body size than honey bees, allowing them to carry more pollen on their bodies. They play a critical role in pollinating fruits with large flowers in tropical regions. It was previously reported that large solitary bees of the genus *Xylocopa* are the main pollinators for yellow passion fruit *P. edulis* (Barrera et al., 2020), and the supply of nests of *Xylocopa frontalis* in crop areas was shown to be effective for the boost of the production and quality of fruits in southeastern Brazil (Toledo et al., 2017). Beyond their commercial value in crop farming, *Xylocopa* bees are also recognized for their ecological importance in tropical rainforest and mangrove forests. In particular, in Malaysia's mangrove forests, *Xylocopa varipuncta* has been identified as a critical pollinator alongside bats and birds and plays a crucial role in carrying pollen for these ecosystems (Hodgkison et al., 2003). Generally, carpenter bees have great potential to increase fruit production and maintain the stability of the ecological system.

In the long-term evolution process, microorganisms harbor in the gut of insects with a mutually interdependent symbiotic relationship. Insects, including Apidae, rely on a mutualistic gut microbial community to digest food, detoxify harmful molecules, provide essential nutrients, protect them from pathogen and parasite invasions, and modulate development and immunity (Engel and Moran, 2013; Douglas, 2015). Eusocial bees, including honey bees (*Apis*) and bumblebees (*Bombus*), harbor host-specific and beneficial microbiota, which play multiple roles in biological activities (Kwong and Moran, 2016; Hammer et al., 2021). However, bee species vary in microbiota composition, including the presence of specialized taxa and the relative bacteria from the environment (McFrederick et al., 2017). The factors that predict this variation in microbiota composition between bee species, as well as the microbial functions that they perform, are poorly understood. However, sociality has been considered a critical driver of gut microbiota evolution in bees (Moran et al., 2019). Here we studied the gut bacterial diversity and community composition of two carpenter bees collected from a tropical rain forest, providing a more comprehensive understanding of the structure of the bacteria community in carpenter bees *Xylocopa*. The results reveal a complex, symbiotic community in the gut of genus *Xylocopa* and provide a molecular basis for understanding the function of the gut microbiota.

Specifically, we determined the bacterial composition of the top 10 most abundant phyla and families of bacteria in two carpenter bees. The results showed that the dominant gut microbiota at the phylum level in two carpenter bees was Firmicutes, Actinobacteria, Proteobacteria, and Bacteroidetes, which were consistent with the previous study in honey bees (Cox-Foster et al., 2007; Khan et al., 2020). Many studies reported that Firmicutes and Proteobacteria were the foremost phyla of the microbiome in the insect gut microbiome, particularly in Hymenoptera (Jeyaprakash et al., 2003). They play a crucial role in processes, such as pectin digestion and mannose degradation, as well as in immune defense against the parasite such as *Nosema bombi* in bumblebees (Martinson et al., 2012; Hammer et al., 2021). The dominant families of the gut microbiome in honey bees are Lactobacillaceae, Bifidobacteriaceae, and Orbaceae. These families perform different functions, such as food digestion and nutrient absorption, which can benefit their host (Powell et al., 2014). In this study, three families dominated almost every sample we tested. As a result, our findings reveal a community composition of two carpenter bees at the phylum and family level that is similar to eusocial bees such as honey bees and bumblebees.

Eusocial bees perform vertically transmitted core gut microorganisms by the fecal–oral route, oral trophallaxis, and contact with hive materials (Powell et al., 2014). Seven bacterial species form the core microbiota of the bumblebee gut: *Snodgrassella, Gilliamella, Schmidhempelia, Bifidobacterium, Bombiscardovia, Bombilactobacillus* (Firm-4), and *Lactobacillus* (Firm-5). These species have been widely studied for their role in food digestion and nutrient absorption by their hosts (Hammer et al., 2021). In contrast, in non-social bee species, even those closely related to social corbiculates, individuals generally harbor microbiomes that are more variable and less distinct. These differences are likely due to the acquisition of microbes being driven by the environment, rather than social factors (McFrederick et al., 2012; Rothman et al., 2020). Moreover, some bee species with incomplete social structures, such as *Megalopta*, also possess gut core bacteria. *Lactobacillus* and *Saccharibacter* were found to be prevalent in 90% of tested adults (Graystock et al., 2017). The previous research has raised the point that environmental transmission appears to be more important than social transmission for *Megalopta* bees (McFrederick et al., 2014). These results reveal that some other factors, rather than social behavior, may be more critical in shaping microbiota structure and specialization. For example, *Xylocopa* species are considered non-classical sociality bees, and their microbiome composition has rarely been studied. However, a recent study has revealed that some incipiently social *Xylocopa* species also have core bacteria in their microbiomes, similar to social bees. In fact, two *Xylocopa* species share a set of core taxa, including *Bombilactobacillus, Bombiscardovia*, and *Lactobacillus*, which were found in most of the individual bees sampled (Handy et al., 2023). Four *Xylocopa* species in central Texas were found to have microbiomes dominated by bacterial taxa that were previously known only in social bees (Holley et al., 2022). In this study, several core gut bacteria of bumblebees were detected in two carpenter bees, which included *Lactobacillus, Bombilactobacillus*, and *Bifidobacterium*, and their abundance in hindguts was significantly higher than in the foregut and midgut. Lactobacillaceae, such as *Lactobacillus* and *Bombilactobacillus*, contain many genes encoding cell membrane proteins and phosphotransferase systems to assist hosts in the absorption and degradation of plant pollen (Kwong et al., 2014). In contrast, Bifidobacteriaceae degrade hexoses via a specific pathway, where the key enzymes are fructose-6-phosphate phosphoketolase (F6PPK) and xylulose phosphoketolase (Bottacini et al., 2012). The symbionts from the Lactobacillaceae and Bifidobacteriaceae were crucial for the health of honey bees, and the findings in carpenter bees were consistent with previous research (Genersch, 2010). Unexpectedly, two *Bombus-*specific core microbes, *Candidatus Schmidhempelia* and *Bombiscardovia*, were abundant in the hindguts of two carpenter bees. *Candidatus Schmidhempelia* is a *Bombus-*specific gamma-proteobacteria, which has been found in 90% of bumblebee guts and has a simplified genome unique to symbiotic bacteria (Martinson et al., 2014). *Bombiscardovia* is a genus of Bifidobacteriaceae originally isolated from *Bombus lapidaries* (Killer et al., 2010). Although the functions of the two genera have not been studied extensively, they are considered beneficial to their hosts. Our results, when compared to the reported gut microbiota of carpenter bees and eusocial bees, show that the genus *Lactobacillus* and *Bifidobacterium* are commonly found in the gut of all studied genus *Xylocopa*. In contrast, the genus *Bombilactobacillus, Gilliamella, Bombiscardovia*, and *Schmidhempelia* are inconsistently distributed in the guts of carpenter bees, and the genus *Snodgrassella* is only found in the guts of eusocial bees. In addition to the core bacteria, the genera *Leuconostoc* and *Apilactobacillus* were found in significantly higher abundance compared to other bacteria. The bacterial taxon known as *Apilactobacillus* was found to be common in the gut and provisions of solitary bees, as well as in the crop of bees (McFrederick et al., 2017). Hypotheses suggest that *Apilactobacillus* may have the ability to inhibit the growth of pathogens or prevent the spoilage of stored pollen (McFrederick et al., 2018).

The specific composition and spatial distribution of the gut microbiota in eusocial bees depend on their functional differentiation (Jeyaprakash et al., 2003). In this study, the differences in bacteria species between the fore-midguts and hindguts of the two carpenter bees were tested by LEfSe analysis. The members of Lactobacillaceae and Bifidobacteriaceae, such as the genera *Bombilactobacillus, Lactobacillus*, and *Bifidobacterium*, were significantly enriched in hindguts. This finding is consistent with the results observed in social bees. In honey bees *A. mellifera*, the hindguts function in fecal storage, reabsorption of nutrients, and enabling the colonization of core bacteria, such as *Bombilactobacillus, Lactobacillus*, and *Bifidobacterium*, which have enriched genes participating in the carbohydrate uptake and metabolism pathways (Kwong and Moran, 2016). The predicted functional pathways, tested by PICRUSt2, in the hindguts of *X. caerulea* and *X. auripennis* were concentrated in carbohydrate and lipid metabolic processes, which corresponded with bacterial species distributed in the hindguts. These results suggest that Lactobacillaceae and Bifidobacteriaceae are dominant in the hindguts and may play a vital role in carbohydrate and lipid metabolism. PICRUSt2 contains an updated and large database of gene families and reference genomes and provides interoperability with any operational taxonomic unit (OTU)-picking or denoising algorithm (Douglas et al., 2020). A large number of studies have used this method to predict the functions of gut microbiota. For example, in the oriental fruit moth *Grapholita molesta*, feeding on different plants can significantly change the functions of gut microbiota (Yuan et al., 2021). However, there are still some limitations when using PICRUSt2 to predict based on a fragment of the V3–V4 region. Future experiments should be designed to use metagenomic or single bacterial genome approaches to conduct more intensive studies.

In eusocial bees, such as honey bees, the core microbiota can be vertically transmitted between workers and larvae through trophallaxis and the fecal–oral pathway (Powell et al., 2014). In contrast, the gut microbes of solitary bees are believed to be acquired from the hive environment and food due to the absence of close social contact (Gilliam et al., 1984). There is mounting evidence that some bacteria taxa, previously known only in social bees, exist in the guts of solitary bees and may have recently transmitted from mother bees to larvae. Previous studies have shown that *Bifidobacterium* isolates from the guts of European *X*. *violacea* were closely related to those of honey bees and bumblebees (Alberoni et al., 2019). In this study, we found that *X. caerulea* and *X. auripennis* shared similar bee-associated bacterial community profiles despite inhabiting different ecological environments. Lactobacillaceae, Orbaceae, and Bifidobacteriaceae were the three main families of gut microbiota in bees, including the genera *Lactobacillus, Apilactobacillus, Bombilactobacillus, Candidatus Schmidhempelia, Bifidobacterium*, and *Bombiscardovia*. Most of the ASVs found in carpenter bees of the three families in this study were closely related to previously identified bacterial taxa, which are widespread in social bees, particularly *Bombus*-specific genera *Candidatus Schmidhempelia* and *Bombiscardovia*. These results suggest that the vertical transmission of bacteria in *Xylocopa* may occur through certain mechanisms.

It is commonly believed that solitary bees exhibit no caring behavior, with mother bees only collecting food for the larvae and leaving the hive before the offspring mature. The gut microorganisms of their offspring are mainly acquired from the environment and from food sources, such as the genera *Megachile* and *Osmia* (Keller et al., 2018; Voulgari-Kokota et al., 2019). However, a previous study of the bee species in the genera *Megalopta* (which contains solitary and social species) found a limited influence of sociality on bacterial composition (McFrederick et al., 2014). This demonstrated that microbiota was not only transmitted by direct social contacts, such as trophallaxis and fecal–oral contact, between concurrent members but also by some non-social behaviors. Furthermore, the vertical transmission of core gut bacteria in two carpenter bee species may be linked to other observed behaviors within the *Xylocopa* genus. In *X. sulcatipes*, different generations of mother bees fight for nest chambers due to competition in a resource-limited environment (Stark, 2010). The usurper drives the host out of the hive or defense against the enemy instead of reproducing and laying new eggs in the used hive, which probably drives microbe–host specificity by contact with old hive materials. In the social bee *A. mellifera*, newly emerged young honey bees chew their way out of cells and consume gut core microbiota that remained on hive surfaces (Martinson et al., 2012). Recent research has confirmed that the transmission of honey bee core hindgut microbiome is facultative and horizontal, with five out of six core hindgut species readily acquired from the built hive structure and natural diet (Anderson et al., 2022). The same route of transmission may exist in the genus *Xylocopa* and result in an accumulation of bacterial species in the guts of young bees from old hive materials. In this study, we collected samples of two carpenter bees in a relatively high population density region where nests are concentrated. Previous studies have predominantly collected solitary bees randomly in the wild, where there is minimal competition pressure and unstable gut microbiota. Thus, our findings indicate that the pressures of nesting and reproduction for *Xylocopa* seem to drive the reuse of old nests and the vertical transmission of gut bacteria, although the life habits of most *Xylocopa* species are poorly studied. The social transmission routes of *Xylocopa* species merit further investigation.

In conclusion, we characterized the gut microbial communities of two carpenter bees and found that some gut bacterial taxa exist in the guts of *X. caerulea* and *X. auripennis*, such as *Candidatus Schmidhempelia* and *Bombiscardovia*, which were closely related to those found in eusocial bees, especially bumblebees. Based on our results, we hypothesize that the gut bacteria of carpenter bees are transmitted from mother bees to larvae by reusing old nests. This study offers novel insight into the structure, distribution, and function of gut symbiotic bacteria in *Xylocopa* species. However, there were still some limitations in our study. Future experiments should be designed to compare the gut microbiota of these two carpenter bees with that of other species in the genus *Xylocopa* and eusocial bees. Moreover, isolating *Candidatus Schmidhempelia* and *Bombiscardovia* from the two *Xylocopa* species and elucidating their important functions using multi-omics will contribute to finding new probiotics that people can use.

## Data availability statement

The datasets presented in this study can be found in online repositories. The names of the repository/repositories and accession number(s) can be found at: NCBI—PRJNA915489.

## Author contributions

WH and YG designed the research and wrote the manuscript. YG, YW, SZ, and DL collected the samples. WH, YG, SW, and YZ performed the experiments and analyzed the data. WH and JG funded this study. All authors contributed to the article and approved the submitted version.

## References

[B1] Alberoni D. Gaggìa F. Baffoni L. Modesto M. M. Biavati B. Di Gioia D. (2019). *Bifidobacterium xylocopae* sp. nov. and *Bifidobacterium aemilianum* sp. nov., from the carpenter bee *(Xylocopa violacea)* digestive tract. Syst. Appl. Microbiol. 42, 205–216. 10.1016/j.syapm.2018.11.005 30551956

[B2] Ali H. Muhammad A. Islam S. U. Islam W. Hou Y. (2018). A novel bacterial symbiont association in the hispid beetle, *Octodonta nipae* (Coleoptera: Chrysomelidae), their dynamics and phylogeny. Microb. Pathog. 118, 378–386. 10.1016/j.micpath.2018.03.046 29596879

[B3] Ali H. Muhammad A. Sanda N. B. Huang Y. Hou Y. (2019). Pyrosequencing uncovers a shift in bacterial communities across life stages of *octodonta nipae* (Coleoptera: Chrysomelidae). *Front. Microbiol*. 10, 466. 10.3389/fmicb.2019.00466 30930872 PMC6424052

[B4] Anderson K. E. Ricigliano V. A. Copeland D. C. Mott B. M. Maes P. (2022). Social interaction is unnecessary for hindgut microbiome transmission in honey bees: the effect of diet and social exposure on tissue-specific microbiome assembly. Microb. Ecol. 10.1007/s00248-022-02025-5 35499645 PMC10167169

[B5] Anderson K. E. Rodrigues P. A. P. Mott B. M. Maes P. Corby-Harris V. (2015). Ecological succession in the honey bee gut: shift in *Lactobacillus* strain dominance during early adult development. Microb. Ecol. 71, 1008–1019. 10.1007/s00248-015-0716-2 26687210

[B6] Barrera W. B. Trinidad K. A. D. Presas J. A. (2020). Hand pollination and natural pollination by carpenter bees (*Xylocopa* spp.) in *Passiflora edulis* Sims. *f. flavicarpa Deg*. (yellow passion fruit). J. Apic. Res. 60, 845–852. 10.1080/00218839.2020.1842580

[B7] Bottacini F. Milani C. Turroni F. Sanchez B. Foroni E. Duranti S.. (2012). *Bifidobacterium asteroides* PRL2011 genome analysis reveals clues for colonization of the insect gut. PLoS ONE 7, e44229. 10.1371/journal.pone.0044229 23028506 PMC3447821

[B8] Brittain C. Kremen C. Klein A. M. (2013). Biodiversity buffers pollination from changes in environmental conditions. Glob. Chang. Biol. 19, 540–547. 10.1111/gcb.12043 23504791

[B9] Cox-Foster D. L. Conlan S. Holmes E. C. Palacios G. Evans J. D. Moran N. A.. (2007). A metagenomic survey of microbes in honey bee colony collapse disorder. Science 318, 283–287. 10.1126/science.1146498 17823314

[B10] Danforth B. N. Cardinal S. Praz C. Almeida E. A. Michez D. (2013). The impact of molecular data on our understanding of bee phylogeny and evolution. Annu. Rev. Entomol. 58, 57–78. 10.1146/annurev-ento-120811-153633 22934982

[B11] de Farias-Silva F. J. Freitas B. M. (2021). Thermoregulation in the large carpenter bee *Xylocopa frontalis* in the face of climate change in the Neotropics. Apidologie 52, 341–357. 10.1007/s13592-020-00824-8

[B12] Douglas A. E. (2015). Multiorganismal insects: diversity and function of resident microorganisms. Annu. Rev. Entomol. 60, 17–34. 10.1146/annurev-ento-010814-020822 25341109 PMC4465791

[B13] Douglas G. M. Maffei V. J. Zaneveld J. R. Yurgel S. N. Brown J. R. Taylor C. M.. (2020). PICRUSt2 for prediction of metagenome functions. Nat. Biotechnol. 38, 685–688. 10.1038/s41587-020-0548-6 32483366 PMC7365738

[B14] Engel P. Kwong W. K. McFrederick Q. Anderson K. E. Barribeau S. M. Chandler J. A.. (2016). The bee microbiome: impact on bee health and model for evolution and ecology of host-microbe interactions. MBio 7, e02164–15. 10.1128/mBio.02164-15 27118586 PMC4850275

[B15] Engel P. Moran N. A. (2013). The gut microbiota of insects - diversity in structure and function. FEMS Microbiol. Rev. 37, 699–735. 10.1111/1574-6976.12025 23692388

[B16] Genersch E. (2010). Honey bee pathology: current threats to honey bees and beekeeping. Appl. Microbiol. Biotechnol. 87, 87–97. 10.1007/s00253-010-2573-8 20401479

[B17] Gilliam M. Buchmann S. L. Lorenz B. J. (1984). Microbial flora of the larval provisions of the solitary bees, *Centris pallida* and *Anthophora* sp. Apidologie 15, 1–10. 10.1051/apido:19840101

[B18] Graystock P. Rehan S. M. McFrederick Q. S. (2017). Hunting for healthy microbiomes: determining the core microbiomes of *Ceratina, Megalopta*, and *Apis* bees and how they associate with microbes in bee collected pollen. Conserv. Genet. 18, 701–711. 10.1007/s10592-017-0937-7

[B19] Hammer T. J. Le E. Martin A. N. Moran N. A. (2021). The gut microbiota of bumblebees. Insect. Soc. 68, 287–301. 10.1007/s00040-021-00837-1 35342195 PMC8956082

[B20] Handy M. Y. Sbardellati D. L. Yu M. Saleh N. W. Ostwald M. M. Vannette R. L. (2023). Incipiently social carpenter bees (*Xylocopa*) host distinctive gut bacterial communities and display geographical structure as revealed by full-length PacBio 16S rRNA sequencing. Mol. Ecol. 32, 1530–1543. 10.1111/mec.16736 36239475

[B21] He C. Niu Z. Luo A. Zhu C. Wu Y. (2013). In-nest ethology and mating strategies of the carpenter bees *Xylocopa* spp. (Hymenoptera: Apidae). Acta Entomol. Sin. 56, 1047–1054. 10.16380/j.kcxb.2013.09.009

[B22] Hedtke S. M. Patiny S. Danforth B. N. (2013). The bee tree of life: a supermatrix approach to apoid phylogeny and biogeography. BMC Evol. Biol. 13, 138. 10.1186/1471-2148-13-138 23822725 PMC3706286

[B23] Hodgkison R. Balding S. T. Zubaid A. Kunz T. H. (2003). Fruit bats (Chiroptera: Pteropodidae) as seed dispersers and pollinators in a lowland malaysian rain forest. Biotropica 35, 491–502. 10.1111/j.1744-7429.2003.tb00606.x

[B24] Holley J.-.C. Jackson M. N. Pham A. T. Hatcher S. C. Moran N. A. (2022). Carpenter bees (*Xylocopa*) harbor a distinctive gut microbiome related to that of honey bees and bumble bees. Appl. Environ. Microb. 88, e0020322. 10.1128/aem.00203-22 35758673 PMC9275229

[B25] Jeyaprakash A. Hoy M. Allsopp M. H. (2003). Bacterial diversity in worker adults of *Apis mellifera capensis* and *Apis mellifera scutellata* (Insecta: Hymenoptera) assessed using 16S rRNA sequences. J. Invertebr. Pathol. 84, 96–103. 10.1016/j.jip.2003.08.007 14615218

[B26] Jones J. C. Fruciano C. Hildebrand F. Al Toufalilia H. Balfour N. J. Bork P.. (2017). Gut microbiota composition is associated with environmental landscape in honey bees. Ecol. Evol. 8, 441–451. 10.1002/ece3.3597 29321884 PMC5756847

[B27] Junqueira C. N. Augusto S. C. (2016). Bigger and sweeter passion fruits: effect of pollinator enhancement on fruit production and quality. Apidologie 48, 131–140. 10.1007/s13592-016-0458-2

[B28] Junqueira C. N. Hogendoorn K. Augusto S. C. (2012). The use of trap-nests to manage carpenter bees (Hymenoptera: Apidae: Xylocopini), pollinators of passion fruit (Passifloraceae: *Passiflora edulis* f. *flavicarpa*). Ann. Entomol. Soc. Am. 105, 884–889. 10.1603/AN12061

[B29] Keasar T. Sadeh A. Shilo M. Ziv Y. (2007). Social organization and pollination efficiency in the carpenter bee *Xylocopa pubescens*. Entomol. Gener. 29, 225–236. 10.1127/entom.gen/29/2007/225

[B30] Keller A. Brandel A. Becker M. C. Balles R. Abdelmohsen U. R. Ankenbrand M. J.. (2018). Wild bees and their nests host *Paenibacillus* bacteria with functional potential of avail. Microbiome 6, 229. 10.1186/s40168-018-0614-1 30579360 PMC6303958

[B31] Kešnerová L. Emery O. Troilo M. Liberti J. Erkosar B. Engel P. (2019). Gut microbiota structure differs between honeybees in winter and summer. ISME J. 14, 801–814. 10.1038/s41396-019-0568-8 31836840 PMC7031341

[B32] Khan K. A. Al-Ghamdi A. A. Ghramh H. A. Ansari M. J. Ali H. Alamri S. A.. (2020). Structural diversity and functional variability of gut microbial communities associated with honey bees. Microb. Pathog. 138, 103793. 10.1016/j.micpath.2019.103793 31626917

[B33] Killer J. Kopecny J. Mrazek J. Havlik J. Koppova I. Benada O.. (2010). *Bombiscardovia coagulans* gen. nov., sp. nov., a new member of the family Bifidobacteriaceae isolated from the digestive tract of bumblebees. Syst. Appl. Microbiol. 33, 359–366. 10.1016/j.syapm.2010.08.002 20950979

[B34] Kumar S. Stecher G. Li M. Knyaz C. Tamura K. (2018). MEGA X: Molecular evolutionary genetics analysis across computing platforms. Mol. Biol. Evol. 35, 1547–1549. 10.1093/molbev/msy096 29722887 PMC5967553

[B35] Kwong W. K. Mancenido A. L. Moran N. A. (2014). Genome sequences of *Lactobacillus* sp. strains wkB8 and wkB10, members of the Firm-5 Clade, from honey bee guts. Genome Announc. 2, e01176-14. 10.1128/genomeA.01176-14 25395644 PMC4241670

[B36] Kwong W. K. Moran N. A. (2016). Gut microbial communities of social bees. Nat. Rev. Microbiol. 14, 374–384. 10.1038/nrmicro.2016.43 27140688 PMC5648345

[B37] Lee K. V. Steinhauer N. Rennich K. Wilson M. E. Tarpy D. R. Caron D. M.. (2015). A national survey of managed honey bee 2013–2014 annual colony losses in the USA. Apidologie 46, 292–305. 10.1007/s13592-015-0356-z

[B38] Lockstone H. E. (2011). Exon array data analysis using Affymetrix power tools and R statistical software. Brief. Bioinform. 12, 634–644. 10.1093/bib/bbq086 21498550 PMC3220870

[B39] Martinson V. G. Magoc T. Koch H. Salzberg S. L. Moran N. A. (2014). Genomic features of a bumble bee symbiont reflect its host environment. Appl. Environ. Microb. 80, 3793–3803. 10.1128/AEM.00322-14 24747890 PMC4054214

[B40] Martinson V. G. Moy J. Moran N. A. (2012). Establishment of characteristic gut bacteria during development of the honeybee worker. Appl. Environ. Microbiol. 78, 2830–2840. 10.1128/AEM.07810-11 22307297 PMC3318792

[B41] McFrederick Q. S. Thomas J. M. Neff J. L. Vuong H. Q. Russell K. A. Hale A. R.. (2017). Flowers and wild megachilid bees share microbes. Microb. Ecol. 73, 188–200. 10.1007/s00248-016-0838-1 27592345

[B42] McFrederick Q. S. Vuong H. Q. Rothman J. A. (2018). *Lactobacillus micheneri* sp. nov., *Lactobacillus timberlakei* sp. nov. and *Lactobacillus quenuiae* sp. nov., lactic acid bacteria isolated from wild bees and flowers. Int. J. Syst. Evol. Microbiol. 68, 1879–1884. 10.1099/ijsem.0.002758 29648528

[B43] McFrederick Q. S. Wcislo W. T. Hout M. C. Mueller U. G. (2014). Host species and developmental stage, but not host social structure, affects bacterial community structure in socially polymorphic bees. FEMS Microbiol. Ecol. 88, 398–406. 10.1111/1574-6941.12302 24579829

[B44] McFrederick Q. S. Wcislo W. T. Taylor D. R. Ishak H. D. Dowd S. E. Mueller U. G. (2012). Environment or kin: whence do bees obtain acidophilic bacteria? Mol. Ecol. 21, 1754–1768. 10.1111/j.1365-294X.2012.05496.x 22340254

[B45] Michener C. (2000). The Bees of the World, 2nd ed. Baltimore, MA: Johns Hopkins University Press.

[B46] Moran N. A. Ochman H. Hammer T. J. (2019). Evolutionary and ecological consequences of gut microbial communities. Annu. Rev. Ecol. Evol. Syst. 50, 451–475. 10.1146/annurev-ecolsys-110617-062453 32733173 PMC7392196

[B47] Powell J. E. Martinson V. G. Urban-Mead K. Moran N. A. Goodrich-Blair H. (2014). Routes of acquisition of the gut microbiota of the honey bee *Apis mellifera*. Appl. Environ. Microbiol. 80, 7378–7387. 10.1128/AEM.01861-14 25239900 PMC4249178

[B48] Ribière C. Hegarty C. Stephenson H. Whelan P. O'Toole P. W. (2019). Gut and whole-body microbiota of the honey bee separate thriving and non-thriving hives. Microb. Ecol. 78, 195–205. 10.1007/s00248-018-1287-9 30467713

[B49] Rothman J. A. Cox-Foster D. L. Andrikopoulos C. McFrederick Q. S. (2020). Diet breadth affects bacterial identity but not diversity in the pollen provisions of closely related polylectic and oligolectic bees. Insects 11, 645. 10.3390/insects11090645 32962223 PMC7564857

[B50] Stark R. E. (2010). Cooperative nesting in the multivoltine large carpenter bee *Xylocopa sulcatipes* Maa (Apoidea: Anthophoridae): do helpers gain or lose to solitary females? Ethology 91, 301–310. 10.1111/j.1439-0310.1992.tb00871.x

[B51] Subta P. Yodsuwan P. Yongsawas R. In-On A. Warrit N. Panha S.. (2020). Bacterial communities in three parts of intestinal tracts of carpenter bees (*Xylocopa tenuiscapa*). *Insects* 11, 497. 10.3390/insects11080497 32756386 PMC7469164

[B52] Toledo J. A. M. Junqueira C. N. Augusto S. C. Arias M. C. Brito R. M. (2017). Accessing the genetic content of *Xylocopa frontalis* bees (Apidae, Xylocopini) for sustainable management in pollination services of passion fruit. Apidologie 48, 795–805. 10.1007/s13592-017-0524-4

[B53] Voulgari-Kokota A. Grimmer G. Steffan-Dewenter I. Keller A. (2019). Bacterial community structure and succession in nests of two megachilid bee genera. FEMS Microbiol. Ecol. 95. 10.1093/femsec/fiy218 30371906

[B54] Wu Y. (2000). Fauna Sinica. Insecta, Vol. 20, Hymenoptera: Melittiae Apidae. Beijing: Science Press.

[B55] Yamamoto M. Junqueira C. N. Barbosa A. A. A. Augusto S. C. Oliveira P. E. (2014). Estimating crop pollinator population using mark–recapture method. Apidologie 45, 205–214. 10.1007/s13592-013-0238-1

[B56] Yuan X. Zhang X. Liu X. Dong Y. Yan Z. Lv D.. (2021). Comparison of gut bacterial communities of *Grapholita molesta* (Lepidoptera: Tortricidae) reared on different host plants. Int. J. Mol. Sci. 22, 6843. 10.3390/ijms22136843 34202141 PMC8268091

[B57] Zhang S. Shu J. Xue H. Zhang W. Zhang Y. Liu Y.. (2020). The gut microbiota in camellia weevils are influenced by plant secondary metabolites and contribute to saponin degradation. mSystems 5, e00692-19. 10.1128/mSystems.00692-19 32184361 PMC7380582

[B58] Zheng H. Powell J. E. Steele M. I. Dietrich C. Moran N. A. (2017). Honeybee gut microbiota promotes host weight gain via bacterial metabolism and hormonal signaling. Proc. Natl. Acad. Sci. U.S.A. 114, 4775–4780. 10.1073/pnas.1701819114 28420790 PMC5422775

[B59] Zheng H. Steele M. I. Leonard S. P. Motta E. V. S. Moran N. A. (2018). Honey bees as models for gut microbiota research. Lab Animal 47, 317–325. 10.1038/s41684-018-0173-x 30353179 PMC6478020

