# Supplementary material for: Xylocopa caerulea and Xylocopa auripennis harbor a homologous gut microbiome related to that of eusocial bees
Source: Front Microbiol. 2023 May 17;14:1124964. doi: 10.3389/fmicb.2023.1124964 (PMC10229870; doi:10.3389/fmicb.2023.1124964)
Supplement: Supplementary file 1 [file Table_1.docx]

**Supplementary material**

*Xylocopa caerulea* and *Xylocopa auripennis* harbor a homologous gut microbiome related to that of eusocial bees

Yifan Gu^1,2,3, †^, Wensu Han^1,4, †, *^, Yuquan Wang^5^, Danlei Liang^3^, Jinglin Gao^1,4, *^, Yihai Zhong^1,4^, Shan Zhao^1,4^, Shijie Wang^1,4^

^1^ Environment and Plant Protection Institute, Chinese Academy of Tropical Agricultural Sciences, Haikou, China

^2^ Sanya Institute of China Agricultural University, Sanya, China;

^3^ Department of Entomology, College of Plant Protection, China Agricultural University, Beijing, China

^4^ Bee Industry Technology Research Center, Chinese Academy of Tropical Agricultural Sciences, Haikou, China

^5^ College of Plant Protection, Hainan University, Haikou, China

*** Correspondence:**

Wensu Han

[hwswill8@126.com](mailto:hwswill8@126.com)

Jinglin Gao

[jinglin.g@163.com](mailto:jinglin.g@163.com)

^†^These authors have contributed equally to this work.

# Supplementary Data

**Table S1** Summary of sequence statistics for the Illumina HiSeq runs of all samples.

| Sample | Raw Tags | Raw Bases | Valid  Tags | Valid  Bases | Valid% | Q20(%) | Q30(%) | GC (%) |
| --- | --- | --- | --- | --- | --- | --- | --- | --- |
| Xa_FMG1 | 82612 | 41.31M | 78990 | 33.16M | 95.62 | 97.25 | 92.28 | 51.11 |
| Xa_HG1 | 80287 | 40.14M | 65281 | 27.31M | 81.31 | 97.22 | 92.25 | 52.05 |
| Xa_FMG2 | 82528 | 41.26M | 70218 | 29.84M | 85.08 | 96.64 | 91.02 | 50.3 |
| Xa_HG2 | 81194 | 40.60M | 70849 | 29.66M | 87.26 | 97.34 | 92.54 | 52.79 |
| Xa_FMG3 | 82719 | 41.36M | 77067 | 32.87M | 93.17 | 97.33 | 92.41 | 50.08 |
| Xa_HG3 | 80216 | 40.11M | 70142 | 29.92M | 87.44 | 96.75 | 91.27 | 49.93 |
| Xa_FMG4 | 82152 | 41.08M | 71951 | 30.22M | 87.58 | 97.22 | 92.36 | 50.56 |
| Xa_HG4 | 83649 | 41.82M | 69801 | 29.18M | 83.45 | 97.3 | 92.58 | 52.64 |
| Xa_FMG5 | 80666 | 40.33M | 73910 | 31.48M | 91.62 | 97.18 | 91.98 | 50.1 |
| Xa_HG5 | 82455 | 41.23M | 71271 | 30.43M | 86.44 | 97.2 | 92.16 | 50.97 |
| Xa_FMG6 | 86751 | 43.38M | 79111 | 32.43M | 91.19 | 97.41 | 92.64 | 54.2 |
| Xa_HG6 | 82616 | 41.31M | 69009 | 29.14M | 83.53 | 97.23 | 92.27 | 50.7 |
| Xc_FMG1 | 87645 | 43.82M | 44734 | 18.42M | 51.04 | 97.54 | 93.15 | 54.85 |
| Xc_HG1 | 86096 | 43.05M | 76749 | 31.65M | 89.14 | 97.5 | 92.99 | 53.56 |
| Xc_FMG2 | 83062 | 41.53M | 68158 | 28.76M | 82.06 | 97.04 | 91.97 | 51.03 |
| Xc_HG2 | 82644 | 41.32M | 67057 | 27.98M | 81.14 | 97.46 | 92.97 | 52.79 |
| Xc_FMG3 | 84899 | 42.45M | 74150 | 31.15M | 87.34 | 97.16 | 92.15 | 51.55 |
| Xc_HG3 | 82861 | 41.43M | 70984 | 30.13M | 85.67 | 97.05 | 91.81 | 51.25 |
| Xc_FMG4 | 81547 | 40.77M | 74806 | 31.25M | 91.73 | 97.16 | 91.88 | 50.27 |
| Xc_HG4 | 85737 | 42.87M | 72537 | 30.75M | 84.6 | 97.31 | 92.5 | 49.3 |
| Xc_FMG5 | 85214 | 42.61M | 70355 | 30.00M | 82.56 | 97.16 | 92.22 | 49.62 |
| Xc_HG5 | 87511 | 43.76M | 68367 | 28.83M | 78.12 | 97.21 | 92.32 | 52.06 |
| Xc_FMG6 | 83567 | 41.78M | 68188 | 28.99M | 81.6 | 97.11 | 92.08 | 49.98 |
| Xc_HG6 | 86304 | 43.15M | 71663 | 30.12M | 83.04 | 97.29 | 92.55 | 51.8 |
| Total | 2004932 | — | 1695348 | — | — | — | — | — |


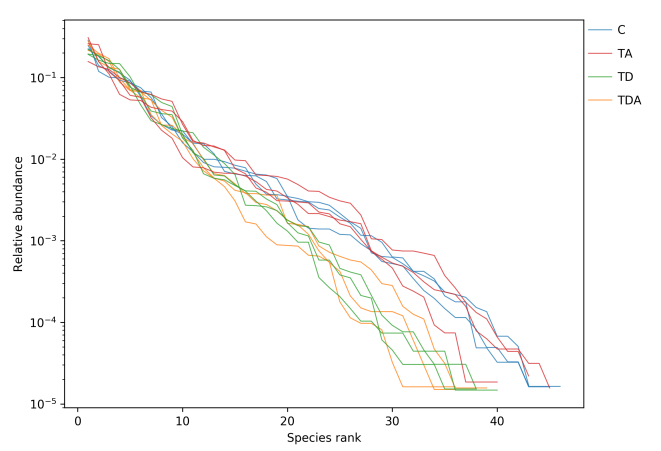


B

C
